# Metabolic activities of five botryticides against *Botrytis cinerea* examined using the Biolog FF MicroPlate

**DOI:** 10.1038/srep31025

**Published:** 2016-08-05

**Authors:** Hancheng Wang, Jin Wang, Licui Li, Tom Hsiang, Maosheng Wang, Shenghua Shang, Zhihe Yu

**Affiliations:** 1 Key Laboratory of Molecular Genetics, Guizhou Academy of Tobacco Science, Guiyang 550081, P.R. China; 2 College of Life Science, Yangtze University, Jingzhou 434025, P.R. China; 3 School of Environmental Sciences, University of Guelph, Guelph, ON, N1G2W1, Canada

## Abstract

Tobacco grey mold caused by *Botrytis cinerea* is an important fungal disease worldwide. Boscalid, carbendazim, iprodione, pyrimethanil and propiconazole are representative botryticides for grey mold management. This research investigated the sensitivities of *B. cinerea* from tobacco to these chemicals using the Biolog FF Microplate. All five chemicals showed inhibitory activity, with average EC_50_ values of 0.94, 0.05, 0.50, 0.61 and 0.31 μg ml^−1^, respectively. *B. cinerea* metabolized 96.8% of tested carbon sources, including 29 effectively and 33 moderately, but the metabolic fingerprints differed under pressures imposed by these botryticides. For boscalid, *B. cinerea* was unable to metabolize many substrates related to tricarboxylic acid cycle. For carbendazim, carbon sources related to glycolysis were not metabolized. For iprodione, use of most carbon substrates was weakly inhibited, and the metabolic profile was similar to that of the control. For propiconazole, no carbon substrates were metabolized and the physiological and biochemical functions of the pathogen were totally inhibited. These findings provide useful information on metabolic activities of these botryticides, and may lead to future applications of the Biolog FF Microplate for examining metabolic effects of other fungicides on other fungi, as well as providing a metabolic fingerprint of *B. cinerea* that could be useful for identification.

*Botrytis cinerea* is a ubiquitous fungus which causes extensive damage under pre- and post harvest conditions, and has been known to infect over 200 plant species worldwide^1^. It causes grey mould on many economically important crops, including lettuce, carrot, tomato, strawberry, rose and tobacco^2^. This fungus has high adaptability due to its polyphagous nature and its diversity of target plant organs (leaf, berry, flower petal and stem)^3^. *B. cinerea* is more destructive on mature and senescent tissues^1^, and losses occur in both field-grown and in greenhouse-grown horticultural crops prior to and after harvest^4^, reaching more than 40% on many crops if no chemical control is applied^5^.

Tobacco (*Nicotiana tabacum* L.) is a leafy, annual, solanaceous plant grown commercially for its leaves. China is the largest single tobacco market, and accounts for around 40% of total global tobacco production and consumption^6^. Tobacco grey mold is important during seedling growth and foliar maturation periods. Tobacco seedlings, stem bases and leaves are all susceptible. The disease occurs frequently in the seedbed and sometimes in the field in southwestern China in two major tobacco production regions: Guizhou and Yunnan. Only older chemical such as carbendazim and mancozeb are used for tobacco grey mould management in China. Newer fungicides such as boscalid and pyrimethanil are not yet registered on tobacco in China.

In recent times, synthetic fungicide use has been the major tool for control of *B. cinerea* worldwide^7^, and during the history of grey mold disease management, many fungicides have been used, such as carbendazim^8^, diethofencarb^9^,^10^, pyrimethanil^11^,^12^, fluazinam^13^, azoxystrobin^14^, iprodione^15^, boscalid^16^, fludioxonil^17^ and propiconazole^18^. Understanding the activity of a chemical against a pathogen at various life cycle stages (especially for conidial germination and mycelial growth) and how it might affect the infection cycles of the pathogen, is critical for disease control. In China, five fungicides with different mode of action against *B. cinerea* have been used for grey mold management on field crops, including boscalid (carboxamide, succinate dehydrogenase inhibitor), carbendazim (benzimidazole), iprodione (dicarboximide), pyrimethanil (anilinopyrimidine) and propiconazole (triazole, sterol biosynthesis inhibitor). However, except for carbendazim, they are not registered in China on tobacco for tobacco grey mold management, and no research has been done on these botryticides nor on tobacco isolates of this pathogen in China.

The Biolog FF MicroPlate was introduced for characterization of filamentous fungi (FF MicroPlate^TM^ Instructions, Biolog, Hayward, CA, USA) using 95 biochemical tests to profile carbon substrate utilization and phenotype^19^, with each well containing substrates that change color with metabolic activity. The carbon substrate utilization help to elucidate the mode of action of each test chemical. There have been a few previous reports of its use for investigating mode of action of fungicides^20^,^21^, and none for these botryticides.

Therefore, the objectives of this current study were to: (i) investigate the *in vitro* activities of five botryticides against *B. cinerea* isolated from tobacco; (ii) examine the metabolic profiling of *B. cinerea* with and without fungicide pressure; and (iii) investigate differences in carbon source utilization of *B. cinerea* under selective pressures of these five different botryticides. The results from this study provide useful information for tobacco grey mould management and also insights into the biochemical effects of these five botryticides.

## Results

### Sensitivity of *B. cinerea* to fungicides

All five chemicals showed strong activity against mycelial growth of five isolates of *B. cinerea* (Table 1).

### Metabolic profiling of *B. cinerea* in the Biolog FF MicroPlate

All five chemicals showed strong activity against mycelial growth of five isolates of *B. cinerea* (Table 1). One highly sensitive isolate, HM4, was chosen for further study. The metabolic abilities of this isolate of *B. cinerea* were tested by using the Biolog FF MicroPlate which includes 95 different carbon sources. The pathogen was able to metabolize 96.8% of the tested carbon sources (92/95 tested, Wells A1-H12). Among them, 29 carbon sources were used efficiently allowing full growth of the fungus. These included N-Acetyl-ß-D-glucosamine (Well A4), L-arabinose (Well A8), D-arabitol (Well A10), arbutin (Well A11), among others. Thirty-three carbon sources were moderately utilized, including ß-cyclodextrin (B2), dextrin (Well B3), D-galacturonic acid (Well B8) among others. Finally, 30 carbon sources were used at a much lower level, including adonitol (Well A6), D-arabinose (Well A8), α-cyclodextrin (Well B1), D-glucosamine (Well B11), among others. The remaining three carbon sources could not be metabolized at all: L-fucose (Well B6), glucuronamide (Well G2) and L-lactic acid (Well F9), resulting in no growth (Table 2).

### Metabolic profiling of *B. cinerea* with exposure to boscalid

**Figure 1:**
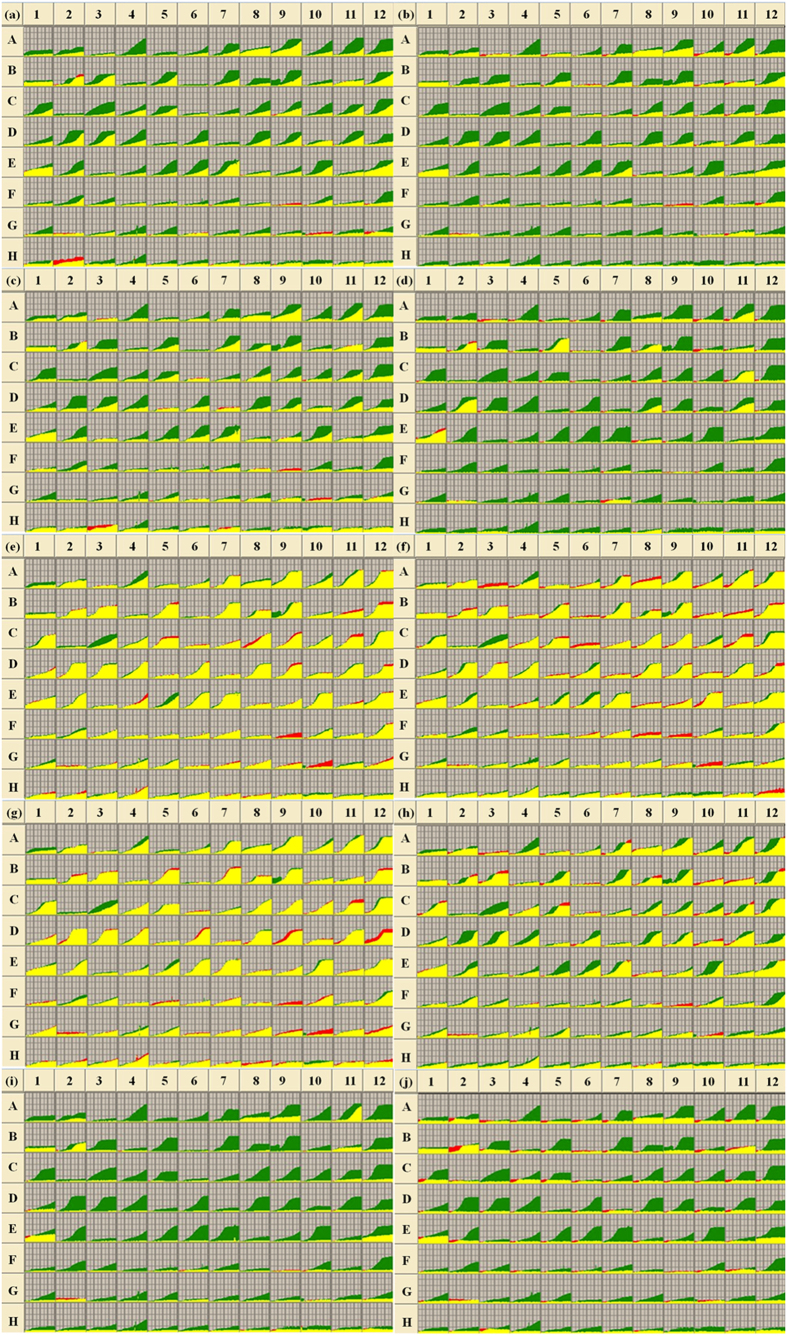
Carbon metabolic fingerprint comparisons of *B. cinerea* between control treatment and treatments of five botryticides. (**a,b**) were comparisons between control and 0.8, and 8 μg ml^−1^ of boscalid, respectively; (**c,d**) were comparisons between control and 0.05, and 1 μg ml^−1^ of carbendazim, respectively; (**e,f**) were comparisons between control and 0.08, and 1 μg ml^−1^ of iprodione, respectively; (**g,h**) were comparisons between control and 1, and 10 μg ml^−1^ of pyrimethanil, respetively; (**i,j**) were comparisons between control and 0.1, and 5 μg ml^−1^ of propiconazole, respectively. Green presented metabolic fingerprint of control treatments; yellow presented common fingerprints of both control and chemical treatments; red presented metabolic fingerprint of chemical treatments.

When treated with boscalid at concentrations of 0.8 or 8 μg ml^−1^, carbon utilization of *B. cinerea* was strongly inhibited. With increasing boscalid concentration, the metabolic ability of *B. cinerea* declined even further. When incubated with boscalid at 0.8 μg ml^−1^, *B. cinerea* was able to metabolized 58 carbon substrates (Table 2). Among these, seven were fully used, including L-arabinose (Well A9), α-D-glucose (Well B12), maltotriose (Well C12), D-mannose (Well D2), D-melezitose (Well D3), sucrose (Well E7) and D-xylose (Well E12). The others were metabolized at a moderate level or a low level (Table 2, Fig. 1(a)). In comparison, in the absence of the fungicide, the utilization of 34 substrates was significantly inhibited (Table 2). When incubated with boscalid at the concentration of 8 μg ml^−1^, 32 substrates were metabolized (Table 2, Table 3). Most carbon substrate utilization was significantly inhibited except for D-arabinose (Well A8), L-arabinose (Well A9), arbutin (Well A11), α-cyclodextrin (Well B1), α-D-glucose (Well B12), D-ribose (Well E1), sucrose (Well E7), D-xylose (Well E12) and L-aspartic acid (Well G11) (Table 4, Fig. 1(b)).

### Metabolic profiling of *B. cinerea* with exposure to carbendazim

Under the pressure of carbendazim at concentrations of 0.05 and 1 μg ml^−1^, the metabolic abilities of *B. cinerea* were significantly inhibited. With the enhancement of carbendazim, the metabolic ability of *B. cinerea* declined greatly. When treated at 0.05 μg ml^−1^ of carbendazim, the pathogen still metabolized 45 substrates (Table 2). Among the metabolized carbons, five were not inhibited at all, including ß-cyclodextrin (Well B2), D-galacturonic acid (Well B8), D-gluconic acid (Well B10), α-methyl-D-glucoside (Well D7) and L-phenylalanine (Well H3). While the utilization of other 47 carbons was inhibited; in between, twenty-seven of them were significantly inhibited (Table 2). When treated at 1 μg ml^−1^ of carbendazim, only 12 out of 95 carbons were used, including D-arabinose (Well A8), arbutin (Well A11), ß-cyclodextrin (Well B2), D-fructose (Well B5), etc. (Table 2, Table 3); while the other substrates were totally inhibited. In comparison with the treatment of carbendazim at 0.05 μg ml^−1^, most carbons utilization were intensively inhibited (Table 4, Fig. 1(d)).

### Metabolic profiling of *B. cinerea* with exposure to iprodione

When incubated with iprodione at the concentration of 0.1 and 5 μg ml^−1^, the metabolic profiling of *B. cinerea* exhibited nearly the same as that of control (Table 2, Fig.1(e,f)). When treated at 0.1 μg ml^−1^ of iprodione, the utilization of four carbons were gently inhibited, including N-acetyl-ß-D-glucosamine (Well A4), D-arabitol (Well A10), D-glucuronic acid (Well C3) and L-sorbose (Well E5). When treated at 5 μg ml^−1^ of iprodione, inhibitions of carbons were still weak (Table 2, Fig. 1(e,f)).

### Metabolic profiling of *B. cinerea* with exposure to pyrimethanil

When incubated at 0.08 and 1 μg ml^−1^ of pyrimethanil, metabolic profiling of *B. cinerea* was weakly influenced by this chemical. Under the pressure of 0.08 μg ml^−1^, *B. cinerea* exhibited nearly the same metabolic profiling as that of control. The utilization of four substrates was weakly inhibited, including N-acetyl-ß-D-glucosamine (Well A4), D-glucuronic acid (Well C3), L-sorbose (Well E5) and bromosuccinic acid (Well F2). While the utilization of five carbons enhanced the growth of the pathogen, including maltose (Well C11), palatinose (Well D9), L-rhamnose (Well D12), D-trehalose (Well F9) and L-asparagine (Well G10) (Fig. 1(g)). Under the pressure of 1 μg ml^−1^ pyrimethanil, the metabolism of around twenty carbon substrates was gently inhibited, including N-Acetyl-ß-D-glucosamine (Well A4), D-arabitol (Well A10), D-glucuronic acid (Well C3), etc. (Table 4, Fig. 1(h)).

### Metabolic profiling of *B. cinerea* with exposure to propiconazole

When treated with propiconazole at the concentration of 1 and 10 μg ml^−1^, the metabolic ability of *B. cinerea* declined greatly (Table 2, Fig. 1(i,j)). With enhancement of propiconazole, less carbon substrates were metabolized. Under the pressure of 1 μg ml^−1^ propiconazole, *B. cinerea* used eight carbons, including D-arabinose (Well A8), L-arabinose (Well A9), arbutin (Well A11), etc. (Table 2, Table 3); the metabolism of other carbon substrates was significantly suppressed (Table 2, Table 4). Under the pressure of 10 μg ml^−1^ propiconazole, *B. cinerea* presented nearly the same metabolic profiling as that of 1 μg ml^−1^ propiconazole except for arbutin (Well A11), which was greatly inhibited (Fig. 1(j)).

## Discussion

Chemical control remains the main way to reduce the incidence of tobacco grey mould caused by *B. cinerea*. Boscalid, carbendazim, iprodione, pyrimethanil and propiconazole are among the most common fungicides to control grey mould. The current study has shown the activities of these chemicals against *B. cinerea* from tobacco, and also provided a carbon metabolic fingerprint of *B. cinerea* that could be useful for identification. The outcome of this analysis has also shown some biochemical effects of these five botryticides from carbon metabonomics.

Although various studies have been performed on *B. cinerea*, this study involved the characterization of the carbon metabolic phenotype of the pathogen *B. cinerea,* revealing significant metabolic diversity. Many carbon compounds could be metabolized. The number of carbon substrates metabolized by *B. cinerea* is higher than that of other pathogens, including *Fusarium kyushuense*^21^. The most informative utilization patterns for carbon sources were saccharides. These compounds are commonly found in many plant organs. They might play a key role in the survival of *B. cinerea* and thus in supporting the pathogenicity of the pathogen.

Boscalid, a fungicide in the carboxamide group, targets succinate dehydrogenase (SDH), which is a functional part of the tricarboxylic cycle and of the mitochondrial electron transport chain^22^. It shows inhibition on the quinine reduction activity^23^. Previous studies indicate that boscalid is highly effective against the diseases caused by various fungi, including *B. cinerea*^24^, *Monilinia fructicola*^25^, *Rhizoctonia solani*^23^ and *Sclerotinia sclerotiorum*^26^. In our tests, *B. cinerea* from tobacco was also sensitive to boscalid. The tricarboxylic acid cycle (TCA) is the final metabolic pathway for glucides, lipids and amino acids, and it is also the hubs to relate the metabolism of these compounds. Under exposure to boscalid in the FF MicroPlate, the substances metabolized in the TCA were inhibited with increasing concentration, including bromosuccinic acid, fumaric acid, α-ketoglutaric acid, D-malic acid, L-malic acid, quinic acid, succinamic acid, succinic acid, L-alaninamide, L-alanine, L-aspartic acid, L-glutamic acid, L-phenylalanine, L-proline, L-pyroglutamic acid, L-serine, L-threonine and putrescine. From this biochemical pathway, succinic acid is one of the metabolites, and its metabolism (Well G4) was strongly inhibited at 0.8 and 8 μg ml^−1^ of boscalid. Due to suppression of SDH activity, and the inhibition of succinic acid metabolism, the whole TCA was affected.

To the best of our knowledge, TCA is the main pathway for the metabolism of glucose, but it is not the only way. When TCA is suppressed, the pentose phosphate pathway (PPP) can be used to metabolize carbohydrates. In our tests, the metabolism of these substrates in TCA was inhibited under the pressure of boscalid, however, some carbon substrates in PPP could be still used and their metabolism was not inhibited, including D-arabinose, L-arabinose, D-ribose, and D-xylose. Thus, it appears that that TCA and PPP were two independent processes during the metabolism of glucide in *B. cinerea*. The biochemical reaction of other substrates inhibited, such as D-glucuronic acid, α-D-lactose, maltitol etc., mainly happened in glycolysis. Thus, it could be supposed that boscalid can also target at some enzymes that needed in glycolysis or TCA, and thus influence the process of glycolysis.

Carbendazim is a systemic benzimidazole fungicide widely used in many countries to control a broad range of fungal diseases of agricultural crops. It inhibits β-tubulin formation during mitosis in susceptible organisms^27^ such as *M. fructicola*, *Penicillium expansum*, *B. cinerea*, and *Tapesia yallundae*^28^. In our tests, isolates of *B. cinerea* from tobacco grey mould were sensitive to carbendazim. The concentrations of carbendazim (0.05 and 1 μg ml^−1^) in metabolic profiling analysis were much higher than the EC_50_ and EC_90_ values of *B. cinerea* in mycelial sensitivity tests on PDA plates. *B. cinerea in vitro* did not grow at the final concentration of 1 μg ml^−1^ carbendazim on PDA plates; however, it still metabolized twelve carbon substrates in the FF MicroPlate analysis, including D-arabinose, arbutin, ß-cyclodextrin, D-fructose, D-galacturonic acid, D-glucosamine, maltose, D-mannose, ß-methyl-D-glucoside, D-raffinose, D-ribose and D-tagatose. In the biochemical processes, most of the carbon substances mentioned above are metabolized in glycolysis which occurs in the cytoplasm; however, carbendazim has its target activity in the cell nucleus. These results indicated that although carbendazim inhibited mitosis of *B. cinerea*, some biochemical pathways related to glycolysis still happened in the cytoplasm; meanwhile, other carbon substrates inhibited by this chemical might directly or indirectly be closely connected to the mitosis of the pathogen. More work could be conducted to verify this hypothesis in a future study.

Iprodione, a dicarboximide fungicide, has a capacity to cause oxidative damage through production of free oxygen radicals^29^. It has been introduced commercially for more than 30 years to control a wide variety of crop diseases, including *B. cinerea*^30^ and *Sclerotinia minor*^31^. One of the target sites of this chemical is histidine kinase (HK)^32^ which is a multifunctional, typically transmembrane, protein of the transferase class. It occurs in intercellular and intracellular spaces and can span cell membranes. Through binding to histidine kinase in the cell, iprodione inhibits signal transduction within the cell, and thus suppress the germination of fungal spores and the growth of fungal mycelium. In our study, the change throughout to concentration of iprodione (0.1 and 5 μg ml^−1^) in the FF MicroPlate analysis were chosen according to the EC_50_ and MIC values in the mycelial growth test. The metabolic profilings of *B. cinerea* in two analyses exhibited nearly the same results as that of control. Iprodione did not affect the carbon substrate metabolism of *B. cinerea* in the cell.

Pyrimethanil is one of anilinopyrimidine fungicides which are a novel family of botryticides introduced in the middle of 1990^12^. It is effective in controlling gray mold and effective against benzimidations and dicarboximide resistance populations in the field, and registered for control of gray mold on vegetable crops worldwide^9^,^33^. It interferes with the biosynthesis of methionine and other amino acids and inhibits the secretion of hydrolytic enzymes, such as pectinases, cellulases and proteases, associated with pathogenesis of *B. cinerea*^34^. In our tests, carbon utilization was not inhibited by 0.08 and 1 μg ml^−1^ of pyrimethanil, likely because pyrimethanil inhibits biosynthesis of methionine, and this biochemical pathway does not directly involve in three important carbon metabolic pathways (glycolysis, TAC and PPP). Additionally, at 1 μg ml^−1^ of pyrimethanil, utilization of 20 substrates were initially inhibited and then metabolized later by *B. cinerea*. More research is needed on the inhibitory effects of pyrimethanil on carbon metabolism. *B. cinerea* might find some new pathways to overcome the biochemical reactions influenced by pyrimethanil. More work should be conducted in the next study to confirm this hypothesis.

Propiconazole is a sterol biosynthesis inhibitor (SBI) fungicide, and is highly effective against gray mold caused by *B. cinerea*^18^. This chemical binds with and inhibits the 14α- demethylase enzyme from demethylating precursors to ergosterol^35^. 14α-demethylase is one of cytochrome P450 enzymes which are a conserved group of proteins that are important in the metabolism of organic substances and the biosynthesis of key steroids, lipids, and vitamins in eukaryotes^36^. 14α-demethylase plays an essential role in mediating membrane permeability in fungi and catalyzes the demethylation of lanosterol to create an important precursor that is eventually converted into ergosterol which constitutes a fundamental component of fungal membranes^37^. In our study with FF MicroPlate, propiconazole inhibited 14α-demethylase activities of the pathogen and prevented the production of ergosterol to stop cellular growth, resulting in no utilization of organic substances. Carbon utilization was greatly inhibited by even 1 μg ml^−1^ of propiconazole. Most substrate utilization was inhibited except for ß-cyclodextrin and D-ribose. These results agreed with the studies reported by other researchers mentioned above. ß-cyclodextrin and D-ribose metabolism may involve pathways which are still able to function even slightly when growth is inhibited by propiconazole.

In conclusion, the results on sensitivity of *B. cinerea* from tobacco to boscalid, carbendazim, iprodione, pyrimethanil and propiconazole provide useful information for the next study of tobacco gray mold management. Carbon substrates are basic building blocks for cells and are important components of cellular activities and biomolecules. These fungicides tested here have known activity against gray mold, but their modes of action at the metabolic level, particularly carbon metabolism, have not all been thoroughly characterized. The results show that metabolism of these substrates is tightly connected with important biochemical pathways in the cell. The metabolic effects of these botryticides against *B. cinerea* add to the information on carbon metabolomics, and provide greater insight for fungicides where mechanisms have not been fully elucidated. In future studies, experimental work is needed to confirm the results observed here on the effects of different fungicides on carbon metabolism.

## Methods

### Pathogen, media and chemical preparation

Five strains of *B. cinerea* (HM1, HM2, HM3, HM4, HM5), with wild-type chemical sensitivity and pathogenicity to tobacco, were collected in 2011 from infected tobacco leaves in a commercial field in Guizhou province, China^38^. These isolates were obtained at least 40 km apart. Monoconidial isolates were obtained, and used for tests. The isolates were grown and maintained on potato dextrose agar (PDA, 200 g L^−1^ potato boiled for half an hour and strained, glucose 20 g L^−1^, agar 16 g L^−1^), in a controlled climate cabinet at 28 °C in darkness. For conidial production, the isolates were placed on PDA at 28 °C, and after 7 days of incubation, plates were moved into a incubator at 15 oC for 48 h. Afterwards, conidia produced on the plates were washed off with sterile water, and filtered through a double-layer of sterile cheesecloth to remove mycelial fragments. The resulting conidial suspension was quantified with a hemacytometer and adjusted to 1 × 10^5^ spores/ml for subsequent use. For long-term storage, 5-mm agar plugs from the leading edge of individual colonies were transferred into several sterile 1.5-ml microcentrifuge tubes containing 1 ml of 30% sterile glycerol, and tubes were stored at −20 °C in darkness.

Technical-grade pyrimethanil (96% active ingredient (a.i.), Jiangsu Gengyun Chemical Co., Ltd, Nanjing), propiconazole (95% a.i., Hubei Zhongliao Chemical Co., Ltd, Wuhan), boscalid (99.9% a.i., Sigma-Aldrich Co. Ltd.,), carbendazim (98% a.i., Zhejiang Xinan Chemical Co., Ltd, Jiande) and iprodione (97.3% a.i., Jiangsu Changlong Chemical Co., Ltd, Nanjing) were used in the assays. Pyrimethanil, propiconazole and boscalid were dissolved in methanol, carbendazim was dissolved in 0.2 M hydrochloric acid solution, and iprodione was dissolved in acetone. The fungicides were all prepared to provide stock solutions containing 10 mg a.i. ml^−1^ and stored at 4 °C in darkness to preserve fungicide activity. Serial dilutions were made with sterile distilled water as required, and were added to autoclaved media (PDA) when cooled to approximately 50 °C. The volumes of methanol, hydrochloric acid and acetone were less than 0.25% of the test solutions. These concentrations did not affect mycelial growth of *B. cinerea* (data not shown). The control always contained the same amount of methanol, hydrochloric acid or acetone as the test samples in the experiments. Filamentous Fungi Inoculating Fluid (FF-IF, catalog # 72106) (containing 2.5 g L^−1^ phytagel and 0.3 g L^−1^ tween 40) and FF MicroPlate test panels (catalog # 1006) containing 95 different carbon sources were purchased from Biolog Inc. (Hayward, CA, USA) and stored at 4 °C until needed.

### Sensitivity of *B. cinerea* to fungicides

To assess the sensitivity of *B. cinerea* to fungicides, a 5-mm mycelial plug was taken from the edge of a 2-day-old colony and placed in the center of a PDA plate amended with fungicide at various concentrations with three replicate plates per isolate by fungicide concentration. The final concentrations tested were as follows: boscalid at 0, 0.31, 0.63, 1.25, 2.50, 5, 10 and 20 μg ml^−1^; carbendazim at 0, 0.0080, 0.016, 0.031, 0.063, 0.13 and 0.25 μg ml^−1^; iprodione at 0, 0.063, 0.13, 0.25, 0.50, 1 and 2 μg ml^−1^; pyrimethanil at 0, 0.13, 0.25, 0.50, 1, 2 and 4 μg ml^−1^; and propiconazole at 0, 0.16, 0.31, 0.63, 1.25, 2.50 and 5 μg ml^−1^. After 5 days of incubation at 28 oC in darkness, the radial growth (colony diameter) of each treatment was measured with the original mycelial plug diameter (5 mm) subtracted from the measurement. For each plate, the average colony diameter measured in two perpendicular directions was used for calculation of the 50% and 90% effective concentration (EC_50_ and EC_90_), which is the fungicide concentration that results in 50% and 90% mycelial growth inhibition (more details on calculations below). The experiments were conducted twice with three replications.

### Metabolic profiling of *B. cinerea* exposed to fungicides

For metabolic profiling of *B. cinerea* with exposure to fungicides, one highly sensitive strain (HM4) was selected and was used here. Conidia were produced on PDA plates as described above, and collected with sterile cotton-tipped applicators from the agar plates, avoiding carryover of nutrients from the agar medium. Conidia together with mycelia were then suspended in distilled water. The FF-IF broth (16 ml broth in 20 ml tubes) were amended with various concentrations of fungicides to obtain final test solutions. The final concentrations tested were as follows: boscalid at 0, 0.8 and 8 μg ml^−1^; carbendazim at 0, 0.05 and 1 μg ml^−1^; iprodione at 0, 0.08 and 1 μg ml^−1^; pyrimethanil at 0, 1 and 10 μg ml^−1^; and propiconazole at 0, 0.1 and 5 μg ml^−1^. Afterwards, each test tube was inoculated with 0.2 ml of spore suspension and mixed gently. One hundred μl of each mixture was then added to each well of the FF plates under aseptic condition. Plates were then incubated in the OmniLog incubator at 28 °C for 7 days with readings taken every 15 minutes. Incubation and recording of phenotypic data were performed in the OmniLog station by capturing digital images of the microarrays and storing turbidity values in a computer file displayed as a kinetic graph. The experiments were conducted twice.

### Data analyses

Data in the sensitivity tests were processed using SIGMASTAT Statistical Software Package (SPSS Science, Chicago). The concentration of fungicide causing 50% (EC_50_) or 90% (EC_90_) reduction in mycelial growth compared to the absence of the fungicide was estimated from the fitted regression line of the log-transformed percentage inhibition plotted against the log-transformed fungicide concentration^39^. Data analysis for metabolic profiling of *B. cinerea* was conducted using Kinetic and Parametric software (Biolog). Phenotypes were determined based on the area under the kinetic curve of dye formation^40^.

## Additional Information

**How to cite this article**: Wang, H. *et al.* Metabolic activities of five botryticides against *Botrytis cinerea* examined using the Biolog FF MicroPlate. *Sci. Rep.* **6**, 31025; doi: 10.1038/srep31025 (2016).

## Figures and Tables

**Table 1:** EC_50_ and EC_90_ values (μg ml^−1^) of five botryticides against mycelial growth of five isolates (HM1 to HM5) of *Botrytis cinerea* a.

Botryticides	HM1		HM2		HM3		HM4		HM5	
	EC_50_	EC_90_	EC_50_	EC_90_	EC_50_	EC_90_	EC_50_	EC_90_	EC_50_	EC_90_
Boscalid	1.41	43.73	1.16	13.74	1.20	29.5	0.50	33.61	0.47	8.45
Carbendazim	0.02	0.33	0.80	1.34	0.10	2.01	0.03	0.11	0.04	0.21
Iprodione	0.48	17.85	0.47	3.30	0.49	11.33	0.11	2.23	0.97	9.65
Pyrimethanil	0.46	40.88	0.81	4.47	2.93	38.18	0.79	18.90	0.37	2.62
Propiconazole	0.21	8.40	0.69	30.72	0.09	10.67	0.13	5.23	0.46	3.54

^a^EC_50_ and EC_90_ values are the concentrations of each chemical causing 50 and 90% reduction in mycelial growth of *Botrytis cinerea* compared with unamended PDA at 28 °C in darkness after 5 days of incubation.

**Table 2:** Carbon substrate utilization profiling of *Botrytis cinerea* under various pressures of five botryticides in Biolog FF Microplate^a^.

Well	Substrate	Fungicides										
		control	Boscalid (μg ml^−1^)		Carbendazim (μg ml^−1^)		Iprodione (μg ml^−1^)		Pyrimethanil (μg ml^−1^)		Propiconazole (μg ml^−1^)	
			0.8	8	0.05	1	0.1	5	0.08	1	1	10
A1	Water	−	−	−	−	−	−	−	−	−	−	−
A2	Tween 80	+	+	−	+	−	+	+	+	+	−	−
A3	N-Acetyl-D-Galactosamine	+	−	−	−	−	+	+	+	+	−	−
A4	N-Acetyl-ß-D-Glucosamine	+++	−	−	−	−	++	++	+++	++	−	−
A5	N-Acetyl-ß-D-Mannosamine	+	−	−	−	−	+	+	+	+	−	−
A6	Adonitol	+	−	−	−	−	+	+	+	+	−	−
A7	Amygdalin	+++	+	−	−	−	+++	+++	+++	+++	−	−
A8	D-Arabinose	+	+	+	+	+	+	+	+	+	+	+
A9	L-Arabinose	+++	+++	++	+++	−	+++	+++	+++	+++	+	+
A10	D-Arabitol	+++	+	+	−	−	++	+++	+++	++	−	−
A11	Arbutin	+++	+	++	++	++	+++	+++	+++	+++	++	−
A12	D-Cellobiose	+++	+	+	+	−	+++	+++	+++	++	−	−
B1	α-Cyclodextrin	+	+	+	+	−	+	+	+	+	+	+
B2	ß-Cyclodextrin	++	++	−	++	+	++	++	++	++	++	++
B3	Dextrin	++	++	−	−	−	++	++	++	++	−	−
B4	i-Erythritol	+	−	−	−	−	+	+	+	+	−	−
B5	D-Fructose	+++	++	+	+	+++	+++	+++	+++	++	−	−
B6	L-Fucose	−	−	−	−	−	−	−	−	−	−	−
B7	D-Galactose	+++	+	+	++	−	+++	+++	+++	++	−	−
B8	D-Galacturonic Acid	++	−	−	+	+	++	++	++	++	−	−
B9	Gentiobiose	+++	++	+	+	−	+++	+++	+++	++	−	−
B10	D-Gluconic Acid	++	−	−	+	−	++	++	++	++	−	−
B11	D-Glucosamine	+	+	+	+	+	+	+	+	+	+	+
B12	α-D-Glucose	+++	+++	−	−	−	+++	+++	+++	++	−	−
C1	α-D-Glucose-1-Phosphate	+++	+	−	−	−	+++	+++	+++	+++	−	−
C2	Glucuronamide	−	−	−	−	−	−	−	−	−	−	−
C3	D-Glucuronic Acid	+++	−	−	−	−	++	++	+++	+	−	−
C4	Glycerol	++	+	−	−	−	++	++	++	++	−	−
C5	Glycogen	+++	+	+	+	−	+++	+++	+++	+++	−	−
C6	m-Inositol	+	−	−	−	−	+	+	+	+	−	−
C7	2-Keto-D-Gluconic Acid	++	−	−	−	−	++	++	++	++	−	−
C8	α-D-Lactose	++	+	+	+	−	++	++	++	++	−	−
C9	Lactulose	+++	+	+	+	−	+++	+++	+++	++	−	−
C10	Maltitol	++	−	−	−	−	++	++	++	++	−	−
C11	Maltose	+++	++	+	+	+++	+++	+++	+++	++	−	−
C12	Maltotriose	+++	+++	+	+	−	+++	+++	+++	++	−	−
D1	D-Mannitol	+++	+	−	−	−	+++	+++	+++	++	−	−
D2	D-Mannose	+++	+++	+	−	+++	+++	+++	+++	+	−	−
D3	D-Melezitose	+++	+++	+	+	−	+++	+++	+++	++	−	−
D4	D-Melibiose	+++	+	+	−	−	+++	+++	+++	++	−	−
D5	α-Methyl-D-Galactoside	+	−	−	−	−	+	+	+	+	−	−
D6	ß-Methyl-D- Galactoside	++	+	−	+	−	++	++	++	++	−	−
D7	α-Methyl-D-Glucoside	+	−	−	+	−	+	+	+	+	−	−
D8	ß-Methyl-D-Glucoside	+++	+	+	+	++	+++	+++	+++	++	−	−
D9	Palatinose	++	++	+	+	−	++	++	++	++	−	−
D10	D-Psicose	+	+	+	+	−	+	+	+	+	−	−
D11	D-Raffinose	+++	++	+	++	+++	+++	+++	+++	+++	−	−
D12	L-Rhamnose	+++	+	+	+	−	+++	+++	+++	+	−	−
E1	D-Ribose	++	++	++	++	+++	++	++	++	++	+	+
E2	Salicin	++	+	−	+	−	++	++	++	++	−	−
E3	Sedoheptulosan	+	−	−	−	−	+	+	+	+	−	−
E4	D-Sorbitol	+	−	−	−	−	+	+	+	+	−	−
E5	L-Sorbose	++	−	−	−	−	+	++	++	+	−	−
E6	Stachyose	+++	+	+	+	−	+++	+++	+++	++	−	−
E7	Sucrose	+++	+++	++	+++	−	+++	+++	+++	+++	−	−
E8	D-Tagatose	+	−	−	−	+	+	+	+	+	−	−
E9	D-Trehalose	++	−	−	−	−	++	++	++	++	-	−
E10	Turanose	+++	++	+	+	−	+++	+++	+++	++	−	−
E11	Xylitol	+	−	+	−	−	+	+	+	+	−	−
E12	D-Xylose	+++	+++	++	++	−	+++	+++	+++	+++	+	+
F1	y-Aminobutyric Acid	+	−	−	−	−	+	+	+	+	−	−
F2	Bromosuccinic Acid	++	+	−	+	−	++	++	++	++	−	−
F3	Fumaric Acid	++	+	−	−	−	++	++	++	++	−	−
F4	ß-Hydroxybutyric Acid	+	−	−	−	−	+	+	+	+	−	−
F5	y- Hydroxybutyric Acid	+	−	−	−	−	+	+	+	+	−	−
F6	p-Hydroxy-phenylacetic Acid	+	−	−	−	−	+	+	+	+	−	−
F7	α-Ketoglutaric Acid	++	+	−	−	−	++	++	++	++	−	−
F8	D-Lactic Acid Methyl Ester	++	−	−	−	−	++	++	++	++	−	−
F9	L-Lactic Acid	−	−	−	−	−	+	+	−	−	−	−
F10	D-Malic Acid	++	+	−	−	−	++	++	++	++	−	−
F11	L-Malic Acid	++	−	−	−	−	++	++	++	++	−	−
F12	Quinic Acid	+++	+	+	−	−	+++	+++	+++	++	−	−
G1	D-Saccharic Acid	+++	−	−	−	−	+++	+++	+++	++	−	−
G2	Sebacic Acid	+	−	−	−	−	+	+	+	+	−	−
G3	Succinamic Acid	+	+	−	−	−	+	+	+	+	−	−
G4	Succinic Acid	++	−	−	−	−	++	++	++	++	−	−
G5	Succinic Acid Mono-Methyl Ester	++	−	−	−	−	++	++	++	++	−	−
G6	N-Acetyl-L-Glutamic Acid	+	−	−	−	−	+	+	+	+	−	−
G7	L-Alaninamide	++	−	−	−	−	++	++	++	++	−	−
G8	L-Alanine	++	−	−	−	−	++	++	++	++	−	−
G9	L-Alanyl-Glycine	++	−	−	−	−	++	++	++	++	−	−
G10	L-Asparagine	++	+	−	−	−	++	++	++	++	−	−
G11	L-Aspartic Acid	++	−	+	−	−	++	++	++	++	−	−
G12	L-Glutamic Acid	++	+	−	+	−	++	++	++	++	−	−
H1	Gycyl-L-Glutamic Acid	++	+	−	+	−	++	++	++	++	−	−
H2	L-Ornithine	++	+	−	+	−	++	++	++	++	−	−
H3	L-Phenylalanine	++	+	+	+	−	++	++	++	++	−	−
H4	L-Proline	++	+	−	+	−	++	++	++	++	−	−
H5	L-Pyroglutamic Acid	+	+	−	+	−	+	+	+	+	−	−
H6	L-Serine	+	+	−	+	−	+	+	+	+	−	−
H7	L-Threonine	+	+	−	+	−	+	+	+	+	−	−
H8	2-Aminoethanol	+	+	−	−	−	+	+	+	+	−	−
H9	Putrescine	+	+	−	+	−	+	+	+	+	−	−
H10	Adenosine	++	+	−	+	−	++	++	++	++	−	−
H11	Uridine	+	+	−	+	−	+	+	+	+	−	−
H12	Adenosine-5′-Monophosphate	+	+	−	+	−	+	+	+	+	−	−

^a^“−”, “+”, “++” and “+++” means that *B. cinerea* could not utilize the tested carbon substrate, utilized poorly, moderately and effectively, respectively in Biolog FF Microplate after 7 days incubation at 28 °C.

**Table 3:** Carbon substrates metabolized by *Botrytis cinerea* at highest tested pressures of five botryticides in Biolog FF Microplate.

Boscalid	Carbendazim	Iprodione and Pyrimethanil			Propiconazole
D-Arabinose	D-Arabinose	Tween 80	Maltose	p-Hydroxy-phenylacetic Acid	D-Arabinose
L-Arabinose	Arbutin	N-Acetyl-D-Galactosamine	Maltotriose	α-Ketoglutaric Acid	L-Arabinose
D-Arabitol	ß-Cyclodextrin	N-Acetyl-ß-D-Glucosamine	D-Mannitol	D-Lactic Acid Methyl Ester	α-Cyclodextrin
Arbutin	D-Fructose	N-Acetyl-ß-D-Mannosamine	D-Mannose	D-Malic Acid	ß-Cyclodextrin
D-Cellobiose	D-Galacturonic Acid	Adonitol	D-Melezitose	L-Malic Acid	D-Glucosamine
α-Cyclodextrin	D-Glucosamine	Amygdalin	D-Melibiose	Quinic Acid	D-Ribose
D-Fructose	Maltose	D-Arabinose	α-Methyl-D-Galactoside	D-Saccharic Acid	D-Xylose
D-Galactose	D-Mannose	L-Arabinose	ß-Methyl-D- Galactoside	Sebacic Acid	
Gentiobiose	ß-Methyl-D-Glucoside	D-Arabitol	α-Methyl-D-Glucoside	Succinamic Acid	
D-Glucosamine	D-Raffinose	Arbutin	ß-Methyl-D-Glucoside	Succinic Acid	
Glycogen	D-Ribose	D-Cellobiose	Palatinose	Succinic Acid Mono-Methyl Ester	
α-D-Lactose	D-Tagatose	α-Cyclodextrin	D-Psicose	N-Acetyl-L-Glutamic Acid	
Lactulose		ß-Cyclodextrin	D-Raffinose	L-Alaninamide	
Maltose		Dextrin	L-Rhamnose	L-Alanine	
Maltotriose		i-Erythritol	D-Ribose	L-Alanyl-Glycine	
D-Mannose		D-Fructose	Salicin	L-Asparagine	
D-Melezitose		D-Galactose	Sedoheptulosan	L-Aspartic Acid	
D-Melibiose		D-Galacturonic Acid	D-Sorbitol	L-Glutamic Acid	
ß-Methyl-D-Glucoside		Gentiobiose	L-Sorbose	Gycyl-L-Glutamic Acid	
Palatinose		D-Gluconic Acid	Stachyose	L-Ornithine	
D-Psicose		D-Glucosamine	Sucrose	L-Phenylalanine	
D-Raffinose		α-D-Glucose	D-Tagatose	L-Proline	
L-Rhamnose		α-D-Glucose-1-Phosphate	D-Trehalose	L-Pyroglutamic Acid	
D-Ribose		D-Glucuronic Acid	Turanose	L-Serine	
Stachyose		Glycerol	Xylitol	L-Threonine	
Sucrose		Glycogen	D-Xylose	2-Aminoethanol	
Turanose		m-Inositol	y-Aminobutyric Acid	Putrescine	
Xylitol		2-Keto-D-Gluconic Acid	Bromosuccinic Acid	Adenosine	
D-Xylose		α-D-Lactose	Fumaric Acid	Uridine	
Quinic Acid		Lactulose	ß-Hydroxybutyric Acid	Adenosine-5′-Monophosphate	
L-Aspartic Acid		Maltitol	y- Hydroxybutyric Acid		
L-Phenylalanine					

**Table 4:** Carbon substrates utilization of *Botrytis cinerea* inhibited at highest tested pressures of four botryticides in Biolog FF Microplate.

Boscalid	Carbendazim		Pyrimethanil		Propiconazole	
Tween 80	Fumaric Acid	Tween 80	D-Trehalose	N-Acetyl-ß-D-Glucosamine	Tween 80	D-Sorbitol
N-Acetyl-D-Galactosamine	ß-Hydroxybutyric Acid	N-Acetyl-D-Galactosamine	Turanose	D-Glucuronic Acid	N-Acetyl-D-Galactosamine	L-Sorbose
N-Acetyl-ß-D-Glucosamine	y- Hydroxybutyric Acid	N-Acetyl-ß-D-Glucosamine	Xylitol	L-Rhamnose	N-Acetyl-ß-D-Glucosamine	Stachyose
N-Acetyl-ß-D-Mannosamine	p-Hydroxy-phenylacetic Acid	N-Acetyl-ß-D-Mannosamine	D-Xylose	D-Mannose	N-Acetyl-ß-D-Mannosamine	Sucrose
Adonitol	α-Ketoglutaric Acid	Adonitol	y-Aminobutyric Acid	L-Sorbose	Adonitol	D-Tagatose
Amygdalin	D-Lactic Acid Methyl Ester	Amygdalin	Bromosuccinic Acid	Turanose	Amygdalin	D-Trehalose
ß-Cyclodextrin	D-Malic Acid	L-Arabinose	Fumaric Acid	Quinic Acid	D-Arabinose	Turanose
Dextrin	L-Malic Acid	D-Arabitol	ß-Hydroxybutyric Acid		L-Arabinose	Xylitol
i-Erythritol	D-Saccharic Acid	D-Cellobiose	y- Hydroxybutyric Acid		D-Arabitol	D-Xylose
D-Galacturonic Acid	Sebacic Acid	α-Cyclodextrin	p-Hydroxy-phenylacetic Acid		Arbutin	y-Aminobutyric Acid
D-Gluconic Acid	Succinamic Acid	Dextrin	α-Ketoglutaric Acid		D-Cellobiose	Bromosuccinic Acid
α-D-Glucose	Succinic Acid	i-Erythritol	D-Lactic Acid Methyl Ester		α-Cyclodextrin	Fumaric Acid
α-D-Glucose-1-Phosphate	Succinic Acid Mono-Methyl Ester	L-Fucose	D-Malic Acid		Dextrin	ß-Hydroxybutyric Acid
D-Glucuronic Acid	N-Acetyl-L-Glutamic Acid	D-Galactose	L-Malic Acid		i-Erythritol	y- Hydroxybutyric Acid
Glycerol	L-Alaninamide	Gentiobiose	Quinic Acid		D-Fructose	p-Hydroxy-phenylacetic Acid
m-Inositol	L-Alanine	D-Gluconic Acid	D-Saccharic Acid		D-Galactose	α-Ketoglutaric Acid
2-Keto-D-Gluconic Acid	L-Alanyl-Glycine	α-D-Glucose	Sebacic Acid		D-Galacturonic Acid	D-Lactic Acid Methyl Ester
Maltitol	L-Asparagine	α-D-Glucose-1-Phosphate	Succinamic Acid		Gentiobiose	D-Malic Acid
D-Mannitol	L-Glutamic Acid	D-Glucuronic Acid	Succinic Acid		D-Gluconic Acid	L-Malic Acid
α-Methyl-D-Galactoside	Gycyl-L-Glutamic Acid	Glycerol	Succinic Acid Mono-Methyl Ester		α-D-Glucose	Quinic Acid
ß-Methyl-D- Galactoside	L-Ornithine	Glycogen	N-Acetyl-L-Glutamic Acid		α-D-Glucose-1-Phosphate	D-Saccharic Acid
α-Methyl-D-Glucoside	L-Proline	m-Inositol	L-Alaninamide		D-Glucuronic Acid	Sebacic Acid
Salicin	L-Pyroglutamic Acid	2-Keto-D-Gluconic Acid	L-Alanine		Glycerol	Succinamic Acid
Sedoheptulosan	L-Serine	α-D-Lactose	L-Alanyl-Glycine		Glycogen	Succinic Acid
D-Sorbitol	L-Threonine	Lactulose	L-Asparagine		m-Inositol	Succinic Acid Mono-Methyl Ester
L-Sorbose	2-Aminoethanol	Maltitol	L-Aspartic Acid		2-Keto-D-Gluconic Acid	N-Acetyl-L-Glutamic Acid
D-Tagatose	Putrescine	Maltotriose	L-Glutamic Acid		α-D-Lactose	L-Alaninamide
D-Trehalose	Adenosine	D-Mannitol	Gycyl-L-Glutamic Acid		Lactulose	L-Alanine
y-Aminobutyric Acid	Uridine	D-Melezitose	L-Ornithine		Maltitol	L-Alanyl-Glycine
Bromosuccinic Acid	Adenosine-5′-Monophosphate	D-Melibiose	L-Phenylalanine		Maltose	L-Asparagine
		α-Methyl-D-Galactoside	L-Proline		Maltotriose	L-Aspartic Acid
		ß-Methyl-D- Galactoside	L-Pyroglutamic Acid		D-Mannitol	L-Glutamic Acid
		α-Methyl-D-Glucoside	L-Serine		D-Mannose	Gycyl-L-Glutamic Acid
		Palatinose	L-Threonine		D-Melezitose	L-Ornithine
		D-Psicose	2-Aminoethanol		D-Melibiose	L-Phenylalanine
		L-Rhamnose	Putrescine		α-Methyl-D-Galactoside	L-Proline
		Salicin	Adenosine		ß-Methyl-D- Galactoside	L-Pyroglutamic Acid
		Sedoheptulosan	Uridine		α-Methyl-D-Glucoside	L-Serine
		D-Sorbitol	Adenosine-5′-Monophosphate		ß-Methyl-D-Glucoside	L-Threonine
		L-Sorbose			Palatinose	2-Aminoethanol
		Stachyose			D-Psicose	Putrescine
		Sucrose			D-Raffinose	Adenosine
					L-Rhamnose	Uridine
					Salicin	Adenosine-5′-Monophosphate
					Sedoheptulosan	
